# Sodium salicylate interferes with quorum-sensing-regulated virulence in chronic wound isolates of *Pseudomonas aeruginosa* in simulated wound fluid

**DOI:** 10.1099/jmm.0.001188

**Published:** 2020-04-22

**Authors:** Erik Gerner, Sofia Almqvist, Maria Werthén, Margarita Trobos

**Affiliations:** ^1^​ Department of Biomaterials, The Sahlgrenska Academy at University of Gothenburg, Gothenburg, Sweden; ^2^​ Mölnlycke Health Care AB, Gothenburg, Sweden; ^3^​ Center for Antibiotic Resistance Research (CARe) at University of Gothenburg, Gothenburg, Sweden; ^4^​ Department of Health Sciences, University West, Trollhättan, Sweden

**Keywords:** *Pseudomonas aeruginosa*, quorum sensing, wound infections, serum, sodium salicylate

## Abstract

**Introduction.:**

An important factor for delayed healing of chronic wounds is the presence of bacteria. Quorum sensing (QS), a cell density-dependent signalling system, controls the production of many virulence factors and biofilm formation in *Pseudomonas aeruginosa *.

**Aim.:**

Inhibition by sodium salicylate (NaSa) of QS-regulated virulence expression was evaluated in QS-characterized clinical wound isolates of *P. aeruginosa,* cultured in serum-containing medium.

**Methodology.:**

Fourteen clinical *P. aeruginosa* strains from chronic wounds were evaluated for the production of QS signals and virulence factors. Inhibition of QS by NaSa in *P. aeruginosa* clinical strains, wild-type PAO1 and QS reporter strains was evaluated using *in vitro* assays for the production of biofilm, pyocyanin, siderophores, alkaline protease, elastase and stapholytic protease.

**Results.:**

Six clinical strains secreted several QS-associated virulence factors and signal molecules and two were negative for all factors. Sub-inhibitory concentrations of NaSa downregulated the expression of the QS-related genes *lasB*, *rhlA* and *pqsA* and reduced the secretion of several virulence factors in PAO1 and clinical strains cultured in serum. Compared to serum-free media, the presence of serum increased the expression of QS genes and production of siderophores and pyocyanin but decreased biofilm formation.

**Conclusions.:**

*
Pseudomonas aeruginosa* from chronic wound infections showed different virulence properties. While very few strains showed no QS activity, approximately half were highly virulent and produced QS signals, suggesting that the targeting of QS is a viable and relevant strategy for infection control. NaSa showed activity as a QS-inhibitor by lowering the virulence phenotypes and QS signals at both transcriptional and extracellular levels.

## Introduction

Chronic or hard-to-heal wounds have a significant impact on quality of life and are associated with a large health-economic burden (2–4 % of health care budgets) [1–3]. Between 1 and 1.5 % of the population in the industrialized world will have a hard-to-heal wound during their lifetime, and infection is an important complication. One reason for wound chronicity is the high prevalence of biofilms (in 80 % of chronic wounds) [4], which results in a drastic increase in bacterial tolerance towards antibiotics [5, 6]. Poor treatment outcome together with the rapid spread of antibiotic resistance, which is estimated to cause more yearly deaths than cancer by 2050 [7], stresses the need for alternative antimicrobial treatment strategies [3, 8].

Two of the most frequently isolated bacterial species in chronic wounds are *Staphylococcus aureus* and *Pseudomonas aeruginosa *, with respective prevalences ranging between 32–94 % and 17–52 % [9, 10]. Particularly, *P. aeruginosa* has been shown to destroy host tissues and is associated with stalled and deteriorating wounds [10–12]. The virulence of the infecting bacteria is believed to play an important role in wound chronicity [13]. Today, it is well established that quorum sensing (QS), a cell and signal density-dependent communication system used by a wide range of micro-organisms, regulates the synthesis of many virulence factors (for a recent review see Lee and Zhang [14]). *Pseudomonas aeruginosa* is globally regulated by at least three QS systems: two *N*-acyl-homoserine lactone (HSL)-mediated quorum-sensing systems, *las* and *rhl*, and the *Pseudomonas* quinolone signal (*pqs*) system (2-heptyl-3-hydroxy-4-quinolone, PQS). The HSL synthases LasI and RhlI mediate the synthesis of the autoinducers N-(3-oxo-dodecanoyl)-L-homoserine lactone (3-OC_12_-HSL) and N-butanoyl-L-homoserine lactone (C_4_-HSL), respectively.

The toxic effects of *P. aeruginosa* are largely mediated by QS-regulated virulence factors, including pyocyanin, elastase and alkaline protease. Pyocyanin is both toxic [15] and involved in biofilm architecture [16, 17]; elastase and alkaline protease degrade host tissues and are involved in iron acquisition from host proteins [18] and host immune evasion [19, 20]. Other virulence-associated factors are rhamnolipids, siderophores and stapholytic proteases. The siderophore pyoverdine plays an essential role in *P. aeruginosa* virulence by capturing iron and regulating the production of exotoxin A and an endoprotease [21]. Rhamnolipids are surfactants with haemolytic and necrotic activities [22], which influence the structure of biofilms [23], and like stapholytic protease [24], are toxic towards a variety of micro-organisms conferring upon *P. aeruginosa* a competitive advantage in the colonization of wound-tissue niches. QS has been found to influence biofilm formation *in vitro* [25, 26]. However, factors other than QS also play a role in biofilm formation since some QS-deficient clinical strains of *P. aeruginosa* were found to form biofilms *in vivo* [27] and *in vitro* with larger surface coverages than the fully QS-competent PAO1 [28]. All of these virulence factors, which are QS-dependent, are major contributors to the ability of this bacterium to cause disease.

Although there is evidence that *P. aeruginosa* clinical isolates from chronic wounds can produce QS signal molecules [13], there is less literature on the characterization of virulence factors. Since QS signals affect virulence-factor production and biofilm development [14], a wound-treatment concept based on QS inhibition would represent an attractive strategy for the prevention and/or treatment of wound infections. Salicylic acid, a metabolite of aspirin, has previously been evaluated as a quorum-sensing inhibitor (QSI) *in vitro* [29, 30]; however, its role as a potential anti-virulence compound to treat human infectious diseases remains to our best knowledge limited to keratitis caused by *P. aeruginosa* [31]. The poor solubility of salicylic acid led us to investigate the potential QS inhibitory effect of the sodium salt of salicylic acid, sodium salicylate (NaSa), which is several hundred-fold more soluble in water than salicylic acid [32, 33].

Screening and evaluation of QSIs are often carried out in simplified *in vitro* test systems based on the cultivation of bacteria in standard media, e.g. minimal medium or lysogeny broth (LB) [34–36]. Although of great importance in evaluating the QS pathways and potential QSIs, these standard media are far from *in vivo* wound conditions, which could result in the risk of limited compound efficacy when QSIs are tested *in vivo* and thus also clinically. A method that represents being one step closer to the evaluation of QSIs in a wound-relevant milieu is to supplement the test medium with serum to better simulate the characteristics of the wound exudate. The total protein content of chronic wound fluid has been determined to be approximately 50 % of the protein content found in serum [37, 38]. Serum and its abundant serum protein albumin are known to interact with and potentially decrease the bioavailability of a wide variety of compounds [39, 40]. Hence, including 50 % serum in the test medium when evaluating QSIs or when studying the phenotypic behaviour of wound pathogens in general increases the relevance of *in vitro* chronic wound models. Taken together, to evaluate the efficacy of potential QSI compounds in the treatment of chronic wounds, it is important to gain more knowledge on the virulence of wound clinical isolates and to use relevant media that resemble the wound bed environment.

The aim of this study was to first characterize 14 clinical *P. aeruginosa* strains isolated from chronic ulcers in terms of their ability to produce QS signal molecules and a range of virulence factors, and then to evaluate the effect of NaSa on the expression of QS genes and production of virulence factors in selected clinical and reference strains grown in serum-containing simulated wound fluid.

## Results

### Characterization of clinical strains isolated from chronic wounds

To add to the current understanding of QS in chronic ulcers and to test the NaSa treatment effect, 14 clinical *P. aeruginosa* strains isolated from chronic wounds were included in the study. All clinical strains were confirmed to belong to the *P. aeruginosa* species using the API kit (bioMérieux SA, Marcy-l’Etoile, France) based on 20 miniature biochemical tests to determine an analytical profile index (API) with scores ≥99.5 % (data not shown). All the strains, including the reference strain PAO1, were then characterized in terms of their ability to produce a number of QS-modulated virulence factors (Table 1), QS signals and biofilm (Table 2). Four clinical strains and PAO1 were positive for all seven tested virulence factors, and two additional strains were positive for five virulence factors. In contrast, two strains were completely negative for all virulence factors evaluated. Of the 14 clinical strains, pyocyanin and siderophores were found in eight strains, stapholytic protease and rhamnolipid in seven strains, alkaline protease in six strains, and elastase and swarming ability were detected in five strains.

**Table 1.** Characterization of the ability of *P. aeruginosa* clinical isolates from chronic wounds to produce virulence factors. All assays were agar-plate-based except for rhamnolipid production, which was evaluated in a glycerol-based liquid medium. Serum-supplemented agar was used for characterization of siderophore production

Strain	1	2	3	4	5	6	7	8	9	10	11	12	13	14	PAO1
Pyocyanin	**+**	−	−	**+**	**+**	**+**	−	**+**	−	−	**+**	**+**	−	**+**	**+**
Siderophores	**+**	−	**+**	**+**	**+**	**+**	−	−	−	−	**+**	**+**	**+**	−	**+**
Alkaline protease	**+**	−	−	**+**	**+**	**+**	−	**+**	−	−	−	**+**	−	−	**+**
Elastase	**+**	−	−	**+**	**+**	**+**	−	−	−	−	−	**+**	−	−	**+**
Stapholytic protease	**+**	**+**	−	−	**+**	**+**	−	**+**	−	−	−	**+**	−	**+**	**+**
Rhamnolipid	**+**	−	−	**+**	**+**	**+**	**+**	**+**	−	−	−	**+**	−	−	**+**
Swarming	**+**	−	−	−	**+**	**+**	−	**+**	−	−	−	**+**	−	−	**+**

**Table 2.** Characterization of clinical *P. aeruginosa* strains from chronic wounds according to their production of the QS signals C_4_-HSL, 3-OC_12_-HSL and PQS and biofilm formation on polystyrene. Supernatants from 24-h-old cultures of each strain in 50 % serum in saline were used for QS-signal analysis using fluorescent reporter strains. Biofilm formation was evaluated on polystyrene surfaces after 48 h culture in 50 % serum in saline using the Calgary biofilm device

Strain	1	2	3	4	5	6	7	8	9	10	11	12	13	14	PAO1
C_4_-HSL	**+**	−	−	**+**	**+**	**+**	−	−	**+**	−	**+**	**+**	**+**	−	**+**
3-OC_12_-HSL	**+**	−	−	**+**	**+**	**+**	**+**	−	−	−	**+**	**+**	**+**	−	**+**
PQS	**+**	−	−	**+**	**+**	**+**	−	−	**+**	−	**+**	**+**	−	−	**+**
Biofilm OD_520_ (STDEV)	1.2 (0.1)	1.4 (0.4)	1.1 (0.6)	1.8 (0.2)	2.4 (0.2)	2.0 (0.7)	1.6 (0.9)	1.1 (0.5)	1.2 (0.5)	1.0 (0.4)	0.9 (0.1)	0.9 (0.4)	1.0 (0.2)	0.8 (0.2)	2.0 (0.9)

Regarding QS signal production, six out of 14 strains were positive for all three QS signals, nine strains and PAO1 were positive for ≥1 QS signal, while in five strains, none of the signals were detected (limits of detection: 0.39 nM, 0.39 nM and 19.5 nM for C_4_-HSL, 3-OC_12_-HSL and PQS, respectively) (Table 2). Concerning their abilities to form biofilm on polystyrene pegs, no clear connection was observed between signal/virulence-factor production and biofilm formation. Three strains (strains 4, 5 and 6) resulted in the highest OD values (similar biofilm biomass as PAO1); the same strains also produced all three QS signals and ≥5 virulence factors. Among the strains with the lowest biofilm biomass, strains 11 and 12 produced the three QS signals, and strain 13 produced two QS signals. In contrast, strains 2 and 7 were moderate biofilm producers with low production of virulence factors.

### Effect of NaSa on QS modulation in PAO1

**Fig. 1.**
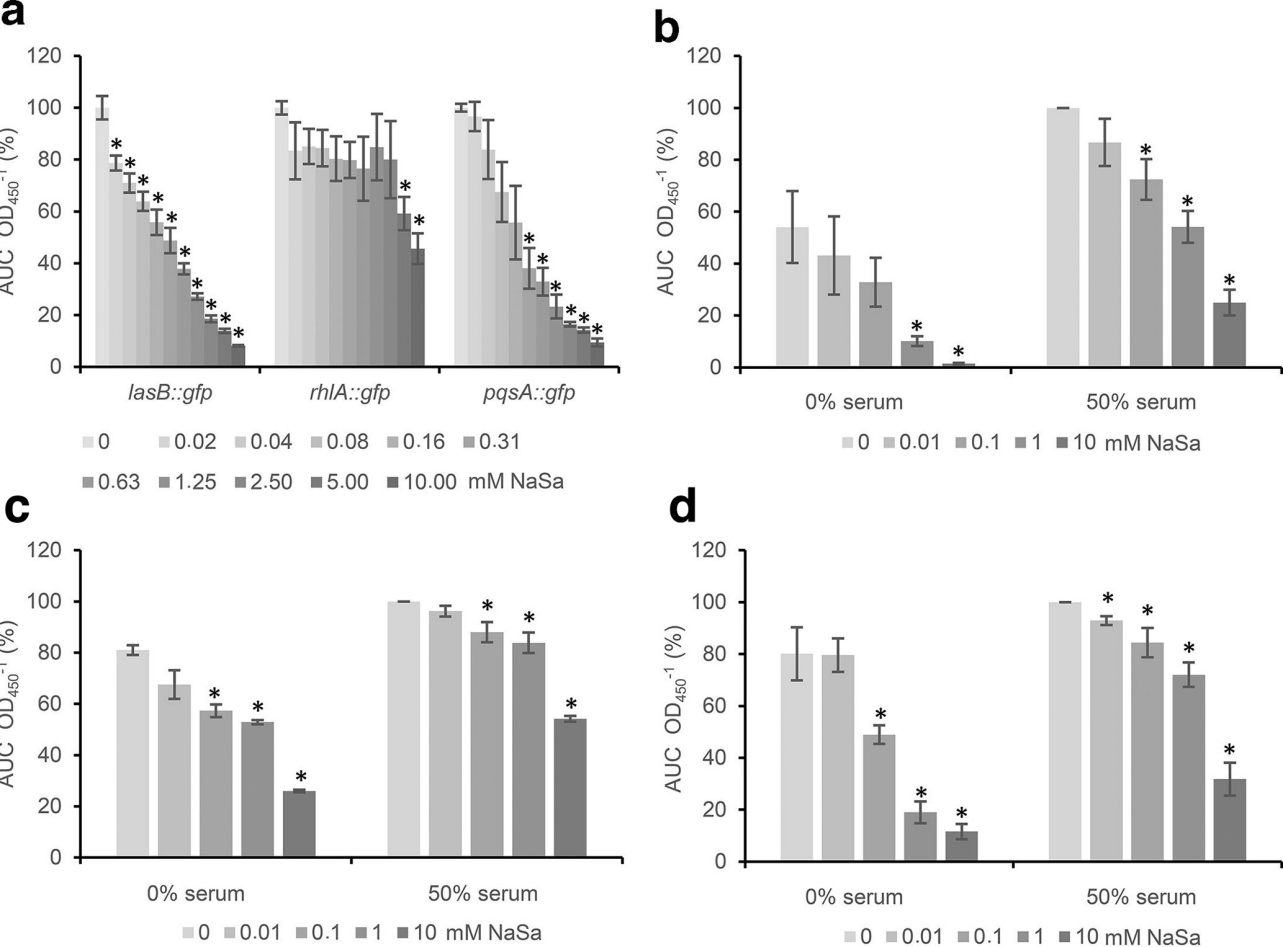
Inhibition of QS by sodium salicylate (NaSa) in *P. aeruginosa *. (a) Concentration-dependent effect of NaSa (0–10 mM) in AB medium on the gene expression of *lasB*, *rhlA* and *pqsA* evaluated with the reporter strains PAO1 *lasB::gfp*, *rhlA::gfp* and *pqsA::gfp*. Effect of NaSa treatment (0–10 mM) in AB media with or without 50 % serum on the expression of (b) *lasB::gfp*, (c) *rhlA::gfp* and (d) *pqsA::gfp*. For each group, the mean area under the curve (AUC) was calculated from each fluorescence intensity curve (baseline- and blank-adjusted) and normalized by OD from three independent experiments. Data in (a) are expressed as % of untreated control in each respective group (100 % corresponds to 4.6×10^7^, 3.6×10^7^ and 5.4×10^6^ fluorescence units for *lasB::gfp*, *rhlA::gfp* and *pqsA::gfp*, respectively). Data in (b–d) are expressed as % of the untreated 50 % serum sample (100 % corresponds to 1.2×10^8^, 7.0×10^7^ and 6.8×10^6^ fluorescence units for *lasB::gfp*, *rhlA::gfp* and *pqsA::gfp*, respectively). Error bars represent ±sd, *N*=3. * Indicates a statistically significant difference compared to untreated 0 mM NaSa group (in the 0 and 50% serum groups, respectively) with a *P*-value <0.05 based on one-way ANOVA Dunnett's post hoc test.

First, the QS modulatory effect of NaSa was evaluated using PAO1-based QS-reporter strains cultured in a commonly used minimal medium [34, 41]. Fluorescent signals from green fluorescent protein (GFP) were acquired and normalized by OD to account for any NaSa-dependent differences in growth rate (Fig. S1, available in the online version of this article). The highest concentration of NaSa tested was below the MIC for all strains (Table S1, available in the online version of this article). Treatment with up to 10 mM NaSa resulted in an overall downregulation of the *las*, *rhl* and *pqs* QS systems in a concentration-dependent manner, as measured by the three reporter strains (Fig. 1a). NaSa treatment was more effective against the *las* (*lasB::gfp*) and *pqs* (*pqsA::gfp*) systems than the *rhl* (*rhlA::gfp*) system. The highest dose of NaSa (10 mM) inhibited the expression levels of *lasB* by 12-fold, of *pqsA* by 11-fold and of *rhlA* by 2-fold. NaSa concentrations of 0.02 mM (for *lasB*), 0.08 mM (for *pqsA*) and 5 mM (for *rhlA*) resulted in a statistically significant reduction in QS expression compared to the control.

Next, to investigate the effect of NaSa on QS in serum-containing media, a series of experiments were performed using the same reporter strains. NaSa significantly reduced the gene expression of the three QS reporter strains, both in the absence and presence of up to 50 % serum, in a concentration-dependent manner (Fig. 1b–d). In 50 % serum, 10 mM NaSa reduced the gene expression of *lasB*, *rhlA* and *pqsA* genes by four-, two- and three-fold, respectively (Fig. 1b–d). In the control group (non-NaSa treated), the addition of 50 % serum resulted in an upregulation in gene expression by 1.85-fold for *lasB*, 1.2-fold for *rhlA* and 1.2-fold for *pqsA* compared to minimal media (no serum) (Fig. 1b–d).

**Fig. 2.**
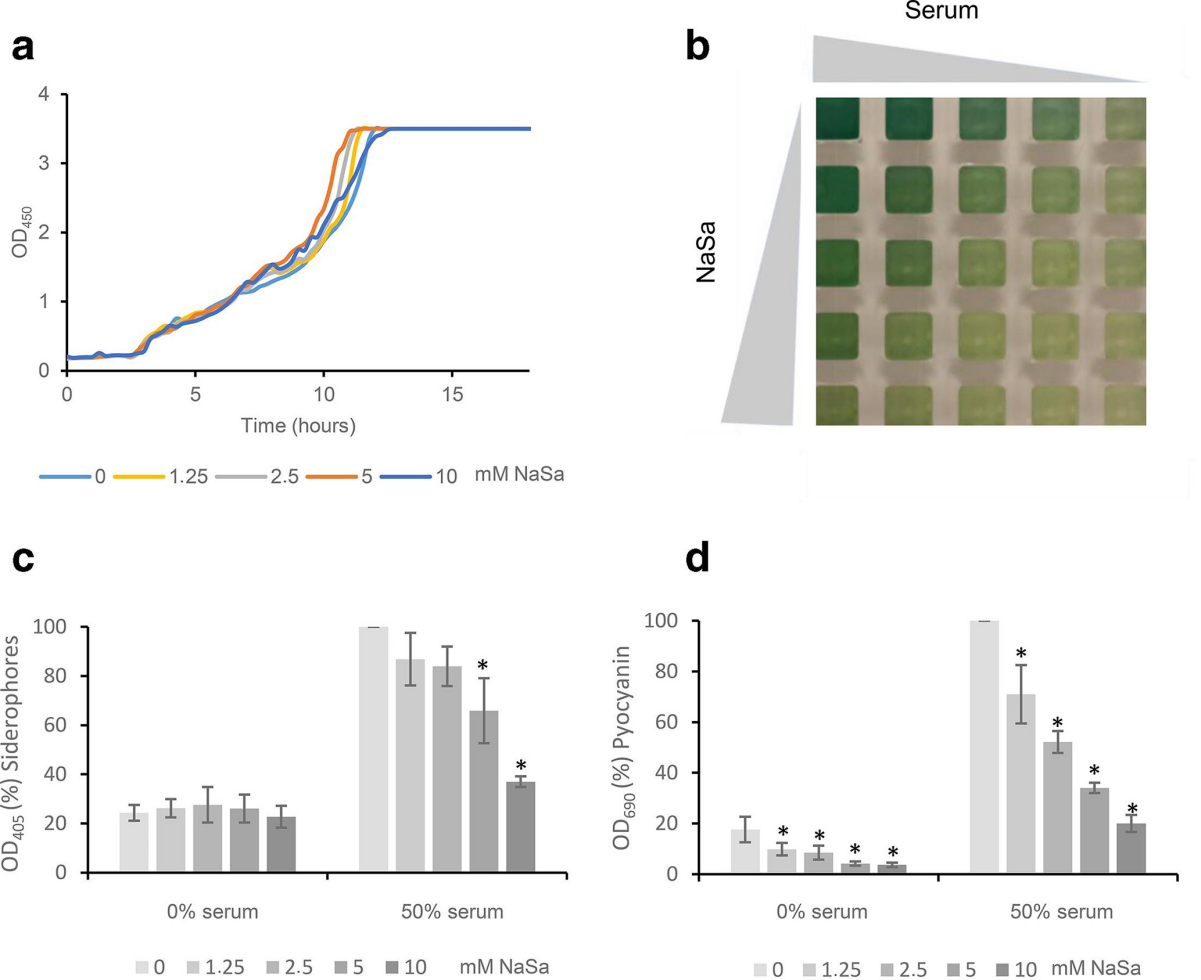
Effect of sodium salicylate (NaSa) on siderophore and pyocyanin production in *P. aeruginosa* PAO1. (a) Growth curves of PAO1 with increasing concentrations of NaSa (0–10 mM) in 50 % serum in AB medium. (b) Visual appearance of supernatants. Effect of NaSa (0–10 mM) and serum (0 and 50 %) on (c) siderophores and (d) pyocyanin production after 3 days of growth. Note: 100% corresponds to OD 6.85 and 1.91 for siderophores and pyocyanin, respectively. Each bar represents the average data from three independent experiments. Error bars represent ±sd. * Indicates a statistically significant difference compared to the untreated 0 mM NaSa group (in the 0 and 50% serum groups, respectively) with a *P*-value <0.05 based on one-way ANOVA Dunnett's post hoc test.

To investigate whether NaSa treatment also influences the synthesis of the actual virulence factors controlled by QS in serum-containing media, wild-type *P. aeruginosa* PAO1 was cultured in minimal AB medium supplemented with increasing concentrations of NaSa and serum using a checkerboard approach. NaSa did not affect the growth rate of PAO1 when grown in 50 % serum (Fig. 2a). In minimal media, NaSa reduced the growth rate (Fig. S2, available in the online version of this article), although after 24 h incubation, there was no difference in c.f.u. counts (approx. 2×10^9^ c.f.u. ml^−1^) between the bacterial cultures taken from the four extreme conditions (NaSa/serum: +/-, -/+, +/+, -/-) represented by the four corners of the checkerboard assay (Fig. 2b).

NaSa treatment of PAO1 in medium supplemented with 50 % serum resulted in a linear decrease in the production of pyocyanin (five-fold decrease for 10 mM NaSa, R^2^=0.82, *P*<0.05) and siderophores (2.7-fold decrease for 10 mM NaSa, R^2^=0.99, *P*<0.001) compared to the control (Fig. 2b–d). The lowest concentration of NaSa tested (1.25 mM) in 50 % serum was enough to significantly reduce pyocyanin production compared to the control, while 5 mM NaSa was required to significantly reduce siderophore production.

In control wells (not NaSa treated), increasing concentrations of serum resulted in a linear increase of siderophore (R^2^=0.99, *P*<0.01) and pyocyanin (R^2^=0.93, *P*<0.01) production (Fig. S3c, d, available in the online version of this article) after 3 days of culture. In 50 % serum, the production of both virulence factors increased approximately six-fold compared to those detected with serum-free media.

A similar effect of serum was observed in the clinical isolates, where 50 % serum significantly increased the secretion of pyocyanin (median 4.3-fold increase) in 6 of 14 strains (Fig. S4a, available in the online version of this article) and siderophores (median 15.7-fold increase) in 11 of 14 strains (Fig. S4b).

In the majority of the strains, 11 out of 14, serum-supplemented culture medium resulted in a significant decrease in biofilm formation on polystyrene surfaces after 24 h (Fig. S4c), with an average reduction of 59 % in biofilm biomass.

### Effect of NaSa on selected virulence factors in strains isolated from wounds

**Fig. 3.**
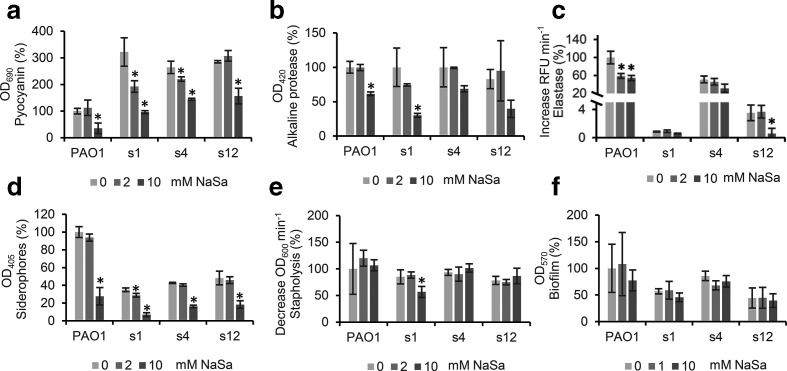
Effect of sodium salicylate (NaSa) on virulence of clinical *P. aeruginosa* strains from chronic wounds (strain 1, 4, 12) and PAO1 after 24 h culture in LB supplemented with 50 % serum and treated with 0 (light grey), 2 (mid grey) or 10 (dark grey) mM NaSa. (a) Pyocyanin production, (b) alkaline protease activity, (c) elastase activity, (d) siderophore production, (e) stapholytic activity and (f) biofilm formation. Pyocyanin production for PAO1 was evaluated after 48 h due to low production after 24 h. Biofilm formation was evaluated in 50 % serum in saline. Data are expressed as % activity or production relative to untreated PAO1 (100 % correspond to increase in fluorescence intensity per min of 9333 for elastase, decrease in OD per min of 0.0012 for stapholysis and to OD readings of 0.53, 0.42, 0.73 and 2.0 for pyocyanin, alkaline protease, siderophores and biofilm, respectively). Error bars represent ±sd, *N*=3. * Indicates a statistically significant difference compared to the untreated 0 mM NaSa group with a *P*-value <0.05 using one-way ANOVA Dunnett's post hoc test.

To investigate the effect of NaSa treatment on clinical isolates, a subset of three highly virulent strains (1, 4 and 12), positive for all investigated QS signals and virulence factors, were treated with 0, 2 or 10 mM NaSa in 50% serum-supplemented medium. These concentrations were chosen based on pyocyanin inhibition in pilot experiments (data not shown). While 2 mM NaSa significantly reduced pyocyanin production in two clinical isolates (strain 1: 40% and strain 4: 16% reduction) (Fig. 3a), 10 mM NaSa was required to significantly reduce alkaline protease activity (strain 1 : 69 % reduction), elastase activity (strain 12 : 83 % reduction) and siderophore production (strain 1 : 80%, strain 4 : 62 % and strain 12 : 62 % reduction) (Fig. 3b–d). Biofilm formation was not affected by NaSa treatment at these concentrations and stapholytic activity was only affected in strain 1 (Fig. 3e, f).

## Discussion

Although some studies include clinical strains, the vast majority of research on QS in *P. aeruginosa* related to clinical issues is carried out using laboratory strains and various reporter strains [42–47]. As suggested by Soukarieh *et al.* [48], two important drawbacks for the further clinical development of QSIs are uncertainty in regard to anti-virulence profiles and efficacy towards *P. aeruginosa* clinical isolates, as well as the lack of standardization of methods used to assess the compounds. A recent study by Cornforth *et al.* [49] further highlights the need of proper evaluation and evidence-based selection of infection models. The authors propose a quantitative framework for model evaluation based on comparing transcriptome data acquired from a target system, such as clinical samples, resulting in an accuracy score [49].

The literature on *P. aeruginosa* QS from a clinical perspective has largely focused on cystic fibrosis [44, 47, 50, 51], whereas studies regarding the QS of clinical *P. aeruginosa* isolates from chronic wounds are limited. To increase the current knowledge, this study included 14 clinical strains of *P. aeruginosa* isolated from patients suffering from infected chronic ulcers and evaluated their ability to produce QS signals and selected virulence factors. PAO1 was included as a virulent reference strain. Although the strain collection was relatively small, this study demonstrates that six out of 14 strains showed a highly virulent phenotype, being positive for at least five out of seven investigated virulence factors. These results are in line with a previous study on chronic wounds where 56 % of the isolates produced two of three investigated virulence factors [46]. In addition, eight of the 14 strains produced at least two QS signals. This high prevalence of signal production was accompanied by increased virulence, as five of the six highly virulent strains were positive for all three QS signals, suggesting that an anti-QS strategy could be a viable treatment option for *P. aeruginosa* strains that cause wound infections.

According to the methodology used in this study, some of the clinical strains do not appear to produce any QS signals but still produce virulence factors (strains 8 and 14), while others produced two QS signals but only ≤1 virulence factor (strains 9 and 13). However, the apparent absence of signals in some strains does not necessarily mean that the strains are signal negative. Signal detection could be influenced by methodological limitations regarding signal stability, sampling time, and the limit of detection of the analytical technique. In fact, the lactone ring of AHL signals undergoes lactonolysis in a pH-dependent manner [52], resulting in signal inactivation. Furthermore, the presence of serum could inactivate as well as degrade signals [53, 54], making the addition of serum relevant to evaluate the potential effect of compounds. Moreover, sampling time also matters [55] and should thus be taken into account. In pilot experiments, different time-points were evaluated to choose the time-point with the highest detection value for some of the factors studied (data not shown). Furthermore, the observation that some of the strains were positive for QS signals but negative for virulence-factor production could be due to mutations in QS receptors. In particular, the LasR receptor is prone to mutation [56], which has been demonstrated in clinical isolates from cystic fibrosis [47] and intubated patients [45]. Hence, non-virulent strains could during establishment of an infection have been virulent, but at some point lost this phenotype. Similarly, strains negative in signal and virulence-factor production *in vitro* could potentially be virulent clinically in close proximity of QS signal-producing strains, since QS signals can be shared between strains [57]. Considering all the above aspects, the *in vivo* pathogenicity of *P. aeruginosa* is multifactorial and dependent on the infectious dose, virulence characteristics of the strains, the ability to produce, sense and respond to QS signals, as well as the host immune response. In this study, any potential relationship between the strains’ virulence and the wound healing outcome could not be studied, since clinical outcome data on the patients were not available.

As further confirmed in this study, *P. aeruginosa* in chronic wounds often expresses QS signals and virulence factors [13, 46], which to a large extent are QS-regulated [14]. Inhibition of QS has been proposed as an alternative treatment strategy for chronic infections [58] and has generated much research interest in the last decades, especially in light of the increasing global threat of antibiotic resistance. Treatment with a QSI could result in reduced expression of virulence factors, aiding the host immune system to clear the infecting pathogen. NaSa is the sodium salt of salicylic acid, a metabolite of the anti-inflammatory and analgesic drug aspirin (acetylsalicylic acid; ASA). This study reports that NaSa reduces QS gene expression in a physiologically relevant milieu, resulting in attenuated secretion of several virulence factors, which could be beneficial clinically. NaSa partially inhibited all three QS systems *las*, *rhl* and *pqs* at the gene expression level in a concentration-dependent manner, using reporter strains for *lasB, rhlA* and *pqsA* grown in medium with and without 50 % serum. Furthermore, a concentration-dependent effect could be demonstrated for the production of virulence factors, as NaSa significantly reduced the secretion of pyocyanin, siderophores, alkaline protease and elastase. A few previous *in vitro* studies have demonstrated the effect of ASA and salicylic acid on QS and/or the production of virulence factors in both Gram-negative and Gram-positive bacteria, e.g. those concerning *P. aeruginosa* [29, 30, 59, 60], *S. aureus* [61–63] and *S. epidermidis* [64]. Interestingly, a clinical study showed that orally administered aspirin reduced the healing time of wounds associated with chronic venous insufficiency, although the mechanism of action was not elucidated [65]. Since NaSa is closely related to ASA and salicylic acid, and because ASA is readily deacetylated *in vivo* [66], it is likely that NaSa could have similar effects. The *in vitro* studies performed on *P. aeruginosa* [29, 30, 59, 60] are all based on laboratory reference strains and two corneal isolates cultured in minimal medium or LB, highlighting the need and relevance of proving treatment efficacy with multiple clinical isolates and serum-supplemented culture conditions to closer resemble the wound environment. Mimicking the conditions where the treatment will be applied, e.g. the wound environment, potentially increases the chance of identifying active compounds with greater potential to be effective *in vivo* and, ultimately, in a clinical setting.

To further evaluate the potential of NaSa as a QSI, its effect was also assessed in three highly virulent clinical strains grown in serum-supplemented media. NaSa decreased the production of four of the five virulence factors tested, indicating that the substance is also effective on clinical *P *. aeruginosa strains. NaSa did not significantly affect the stapholytic activity, except for strain 1. Further, NaSa did not display any effect on the amount of biofilm biomass formed by any of the clinical wound isolates (only three shown here) or the PAO1 strain, when biofilms were grown on the surface of the Calgary biofilm device under dynamic conditions (Fig. 3f).

A drawback of the widely used microtitre plate assay for biofilm quantification using crystal violet is that bacterial aggregates formed in the bulk solution are not considered [67], and it could still be possible that NaSa may affect biofilm aggregation in suspension. It is well established that biofilm is formed in chronic wounds [4, 68] and that aggregated bacteria are abundant in the bulk solution of bacterial cultures [69, 70]. Further studies in our lab will evaluate the effect of NaSa in a three-dimensional biofilm model [67], using confocal laser scanning microscopy and labelling of biofilm matrix components, to evaluate how biofilm composition and architecture may be influenced by NaSa.

Previously, it was shown that ASA decreased biofilm formation on polystyrene in serum-free medium, although a high concentration of 34 mM was required [59]. Similarly, medium with 0.11 mM ASA was shown to reduce the total biofilm biomass produced in a biofilm flow-chamber system using minimal media supplemented with glucose [30]. However, since this is based on subjecting the bacterial cells to a constant flow of ASA-containing medium, it is difficult to determine the total amount of ASA that the cells were exposed to.

Including serum in the test medium drastically reduced *P. aeruginosa* biofilm formation on polystyrene pegs for the majority of clinical isolates after 24 h under dynamic conditions. Serum has previously been shown to reduce early biofilm formation by *P. aeruginosa* on plastic surfaces*,* possibly by increasing twitching motility [71, 72] or because of the low levels of free iron caused by iron-binding proteins such as lactoferrin [73–75].

Furthermore, in control samples without NaSa, supplementing the medium with increasing concentrations of serum resulted in increased QS activation, most notably in the *lasB::gfp* and *pqsA::gfp* reporter strains. Serum also increased pyocyanin and siderophore production in *P. aeruginosa* clinical strains and PAO1. These results agree with a previous study in which serum was shown to increase the expression of the *lasI, rhlA* and *pqsA* genes in *P. aeruginosa,* accompanied by increased virulence-factor production in late stage cultures (16 h) [76]. It was proposed that in the late phase, serum enhances *vfr* expression, resulting in increased levels of the transcription modulator Vfr, which activates the *lasR* gene. This leads to increased 3-OC_12_-HSL production via enhanced *lasI* transcription. Additionally, LasR activates *rhlR/rhlI* transcription, increasing C_4_-HSL levels, and LasR enhances *mvfR* expression, inducing the transcription of the *pqsA-E* operon leading to higher levels of PQS. Since Vfr is a positive regulator of *lasR-* and *rhlR* transcription [77, 78], it could also explain the increased levels of siderophores and pyocyanin in the presence of serum, although *vfr* expression was not evaluated in the present study. In contrast, albumin, a major component of serum, has been shown to decrease *las-* and *rhl-*associated QS, but not that associated with *pqs,* by binding QS signal molecules [53], indicating that serum components other than albumin also play a role in *las* and *rhl*. The serum-dependent increased production of siderophores could also be due to changes in iron concentrations. Studies have shown that low levels of free iron, as in serum due to iron-binding proteins such as transferrin [79, 80], induce siderophore production. It has also been shown that serum increases the virulence of the problematic wound pathogen *S. aureus *, further highlighting the relevance of including serum when studying wound infection *in vitro* [81].

In the present study, a trend was observed in which the inclusion of serum in the test medium decreased the potency of NaSa treatment compared to that achieved in serum-free medium, although only the *las* system was significantly reduced with 10 mM NaSa treatment. It was previously shown that serum components such as albumin interact with a wide variety of pharmaceuticals [40], e.g. aspirin [82, 83]. Importantly, the present results show that NaSa was still effective in medium supplemented with 50 % serum. This indicates that NaSa could potentially be effective as a QSI in the wound environment where serum is a major component.

In conclusion, this study shows that NaSa significantly decreases the gene expression of the *las*, *rhl* and *pqs* QS systems and attenuates the production of pyocyanin, siderophores, alkaline protease and elastase in *P. aeruginosa* wound strains and PAO1 cultured in simulated wound fluid. Serum alone increased QS gene expression of the *las* system and production of pyocyanin and siderophores, while biofilm formation was reduced, stressing the importance of including serum *in vitro* when appropriate.

The characterization performed on the clinical isolates from chronic wounds demonstrated that approximately half of the strains were highly virulent and expressed several QS signals, highlighting the relevance of using QSIs such as NaSa in the clinical setting as an alternative to conventional antibiotics. The observed QS inhibitory effect of NaSa will be further evaluated *in vivo*.

## Methods

### Bacterial strains, culture conditions and chemicals

Fourteen *P. aeruginosa* clinical isolates from chronic leg ulcers, kindly provided by Professor Artur Schmidtchen (Department of Clinical Sciences, Lund University, Sweden) were used in this study. The mutants and reporter strains used were kindly provided by Professor Thomas Bjarnsholt (Costerton Biofilm Center, University of Copenhagen, Denmark) and Professor Yang Liang (Southern University of Science and Technology, China). All chemicals used were purchased from Sigma-Aldrich (USA) if not stated otherwise.

All clinical strains were propagated on 5 % (w/v) horse blood Columbia agar (Medium Department, Clinical Microbiology Lab, Sahlgrenska University Hospital, Sweden). QS reporter strains were cultured in minimal medium (AB medium) consisting of nine parts B-medium (1 mM MgCl_2_, 0.1 mM CaCl_2_, 0.01 mM FeCl_3_) and one part A10-buffer [20 g l^−1^ (NH_4_)_2_SO_4_ (Merck, USA), 60 g l^−1^ Na_2_HPO_4_×2H_2_O, 30 g l^−1^ KH_2_PO_4_ (Acros Organics, USA) and 30 g l^−1^ NaCl] supplemented with 0.5 % (w/v) casamino acids (BD, USA), 0.1 % (w/v) glucose (Merck, USA), and 0.025 % (w/v) thiamine. The AB medium for QS signal- and QS interference reporter strains was supplemented with 10 µg ml^−1^ gentamicin and 100 µg ml^−1^ ampicillin or 60 µg ml^−1^ gentamicin, respectively. All serum used was of foetal calf origin (non-heat inactivated, HyClone, GE Healthcare Life Sciences, USA). The strains used are listed in Table 3.

**Table 3.** List of strains used in this study

Strain name	Characteristic	Reference
From 1 to 14	Clinical wound isolates of *P. aeruginosa *	[96]
PAO1	Reference strain of *P. aeruginosa, Pseudomonas Genetic Stock Center (http:// www.pseudomonas.med.ecu.edu, strain PAO0001)*	
MH155	3-OC12-HSL signal reporter. *Escherichia coli* MT102 with *lasB*::*gfp*(ASV)	[34]
MH2O5	C4-HSL signal reporter. *E. coli* MT102 with *ahyR-ahyI*::*gfp*(ASV)	[91]
∆*pqsC* P*pqsA*::*gfp(ASV)*	PQS signal reporter. PAO1 ∆*pqsC* with *pqsA*::*gfp*(ASV)	[97]
PAO1 P*lasB*::*gfp(ASV)*	*las* reporter. PAO1 with a mini-Tn5 insert containing a translational fusion of the LasR-regulated *lasB* promoter and a gene coding for an unstable version of green fluorescent protein (GFP), gfp(ASV)	[34]
PAO1 *rhlA*::*gfp(ASV)*	*rhl* reporter. PAO1 carrying a pMHRA plasmid containing RhlR-regulated *rhlA::gfp(ASV)* translational fusion inserted into the vector pMH391	[30]
PAO1 *pqsA*::*gfp*	*pqs* reporter. PAO1 carrying the plasmid pAC37 containing a transcriptional fusion of the pqs-regulated *pqsA* promoter and *gfp(ASV)*	[97]
MH694	PAO1 ∆*lasI*. QS-deficient mutant with a deletion of *lasI* gene	[98]
MH698	PAO1 ∆*rhlI*. QS-deficient mutant with a deletion of *rhlI* gene	[35]
∆*pqsC*	PAO1 ∆*pqsC*. QS-deficient mutant with a deletion of *pqsC* gene	[99]

### Characterization of *P. aeruginosa* clinical strains from chronic wounds

#### (i) Virulence characterization

##### Pyocyanin and siderophore production

Overnight cultures of the clinical strains and PAO1 on blood agar were diluted in saline [0.9 % (w/v) NaCl] until OD_546_=0.1 was reached, which corresponds to 10^8^ c.f.u. ml^−1^, and then 100 µl was added to Kings agar A in 24-well plates and 5 µl was added to Pseudomonas F-agar in 9 cm diameter petri dishes for detection of pyocyanin and siderophore (pyoverdine and pyochelin) production [84], respectively. Plates were incubated for 24 h (siderophores) and 48 h (pyocyanin) at 35 °C in a humidified incubator. Visual detection of green colouration in the agar indicated that a strain was positive for pyocyanin production. Fluorescent colonies under UV light were considered positive for siderophore production [85]. At least two of three independent experiments needed to be positive for a strain to be considered a pyocyanin or siderophore producer.

##### Alkaline protease and elastase

The presence of alkaline protease and elastase was detected using modified agar-based assays [86, 87]. Overnight cultures of the clinical isolates and PAO1 on blood agar were diluted to OD_546_=0.1 in saline, and 5 µl were plated on agar plates containing 1.5 % (w/v) tryptic soy agar (TSA, Merck, USA) in 100 mM Tris adjusted to pH=10 and supplemented with 2 % (w/v) skimmed milk powder (Scharlau, Spain) for alkaline protease determination or on plates containing 1.5 % (w/v) TSA and 0.3 % (w/v) insoluble elastin from bovine neck ligaments for elastase determination. The plates were incubated at 35 °C for up to 48 h in a humidified incubator. Strains showing visual signs of degraded milk proteins (24 h) or elastin (48 h) around the colonies were considered positive for alkaline protease and elastase, respectively. Strains with at least two of three positive independent trial results were considered alkaline protease or elastase producers.

##### Swarming

Overnight cultures of the clinical isolates and PAO1 on blood agar were diluted to OD_546_=0.1 in saline, and 2 µl spots were plated on agar containing 1.5 % (w/v) TSA and 2 % (w/v) skimmed milk powder. Swarming behaviour was observed during a period of 48 h of incubation in a humidified incubator at 35 °C. The experiment was repeated three times.

##### Rhamnolipids

Rhamnolipid production was evaluated in a glycerol-based medium [88]. Overnight cultures of the clinical isolates and PAO1 on blood agar were diluted to OD_546_=0.1 in saline. A total of 100 µl of the OD suspension was added to 3 ml medium containing 42 g l^−1^ glycerol, 1.4 g l^−1^ NaNO_3_, 7 g l^−1^ K_2_HPO_4_, 3 g l^−1^ KH_2_PO_4_ and 0.2 g l^−1^ MgSO_4_×7 H_2_O. Rhamnolipids were quantified based on the orcinol assay [89]. In brief, after 6 days of culture at 35 °C and 100 r.p.m., the supernatants were collected by centrifugation at 14 000 ***g*** for 10 min. A volume of 300 µl of supernatant was added to 1.7 ml diethyl ether (VWR, USA), vortexed and centrifuged using a bench centrifuge (iFuge, Neuation, India) at 2000 ***g*** for 1 min. The diethyl ether fraction was collected and evaporated overnight prior to the addition of 300 µl dH_2_O. After vortexing, 100 µl of the rhamnolipid-water solution was added to 900 µl 0.19 % orcinol (w/w) in 53 % (w/w) H_2_SO_4_. The mixture was incubated at 80 °C for 30 min before cooling down at RT. Of this mixture, 200 µl was added to the wells of a 96-well plate (Nunc, Denmark), and the OD was read at 421 nm using a plate reader (FluostarOmega, BMG LABTECH, Germany). Test medium without bacteria, using the same sample preparation, was used as a blank. A strain was considered a rhamnolipid producer if the average OD from three independent experiments differed from that of the blank with statistical significance.

##### Stapholytic activity

LasA protease activity was measured by determining the ability of *P. aeruginosa* culture supernatants to lyse boiled *Staphylococcus aureus* cells [24]. *Staphylococcus aureus* ATCC 25923 was grown in 5 ml TSB at 35 °C and 100 r.p.m. in a shaking incubator (KS4000i, IKA, Germany). The cells were harvested by centrifugation at 4000 ***g*** for 10 min and added to 30 ml of 10 mM Na_2_PO_4_ (dH_2_O) containing 1.5 % (w/v) TSA. The mixture was boiled for 15 min and added to two sterile petri dishes (9 cm ø). After solidification, 5 µl of bacterial suspension of OD_546_=0.1 in saline of each clinical isolate and PAO1 was spotted on each agar plate. The plates were incubated upside down at 35 °C in a humidified incubator for 24 h. Clear zones in the agar around the colonies in at least two out of three independent experiments indicated that a strain was positive for stapholytic activity.

##### Biofilm formation

Biofilm formation was studied using the MBEC P and G Assay (Calgary biofilm device, Innovotech, Canada) and crystal violet [90]. Overnight cultures of the clinical isolates and PAO1 on blood agar were diluted to OD_546_=0.1 in saline supplemented with 50 % (v/v) serum. A volume of 5 µl of each respective strain was added to 145 µl 50 % (v/v) serum-supplemented saline (2×10^7^ c.f.u ml^−1^ final concentration) in the MBEC 96-well plate with polystyrene pegs on the lid. Medium without cells was used as blank. After 48 h incubation at 35 °C and 100 r.p.m. in a humidified shaking incubator, the lid was transferred to a new 96-well plate containing 170 µl per well of 0.1 % (w/v) crystal violet solution and incubated at RT for 15 min. Unbound dye and loosely attached bacterial cells were repeatedly washed off in a water bath until no more dye was visible in the eluent. The lid was air-dried for 30 min before the pegs were immersed in 200 µl 96 % (w/w) ethanol for elution of the dye. After 30 min, the OD was measured at 590 nm. The data are presented as blank-corrected average OD values from three independent experiments, each with three technical replicates.

### (ii) QS signal characterization

QS signal measurements were based on protocols described previously for C_4_-HSL [91], 3-OC_12_-HSL [34] and PQS [92]. Overnight cultures of the clinical isolates on blood agar were diluted to OD_546_=0.2 in 50 % (v/v) serum in saline and were further diluted 1 : 10 in the same medium. A total of 10 ml of bacterial suspension was added to 50 ml Falcon tubes and cultured at 35 °C and 100 r.p.m. for 24 h. Supernatants were collected by centrifugation at 4000 ***g*** for 10 min, sterile filtered and frozen at −80 °C until further analysis. PAO1 was used as a positive control, and mutants negative for signal production were used as negative controls (PAO1 ∆*lasI,* PAO1 ∆*rhlI* and PAO1 ∆*pqsC*). Overnight cultures of the signal reporter strains MH155, MH205 and ∆*pqsC* P*pqsA::gfp*(ASV) were diluted to OD_546_=0.1 in AB medium supplemented with 10 µg ml^−1^ gentamicin and 100 µg ml^−1^ ampicillin. Purified signals [*N*-(3-oxo-dodecanoyl)-L-homoserine lactone ‘3-OC_12_-HSL’], [*N-*butanoyl-L-homoserine lactone ‘C_4_-HSL’ and 2-heptyl-3-hydroxy-4(1 H)-quinolone ‘PQS’] from frozen DMSO stock solutions (1 mg ml^−1^ for 3-OC_12_-HSL and C_4_-HSL or 20 mM for PQS) were serially diluted in 50 % (v/v) serum in saline from 20 µM to 0.2 nM, with pure medium used as a blank. Next, 25 µl of supernatant or purified signal was mixed with 25 µl of each respective signal reporter strain in a black 384-well plate (Greiner Bio-one, Austria) with a clear bottom. The plate was placed in a plate reader, and kinetic measurements of the absorbance (450 nm) and fluorescence (bottom reading, excitation 530/20 and emission at 485/30 filters) were recorded every 15 min for 20 h at 35 °C. The area under the curve (AUC) data of baseline- and blank-adjusted fluorescence intensity (FI) (OD normalized) from three independent experiments, with at least two technical replicates, were used to characterize the clinical strains in terms of signal production. As a cut-off value for signal production characterization, strains with AUC data within the standard curve and above that of the signal deficient mutant were considered positive for QS signal production.

### Effect of NaSa on PAO1 QS activation and virulence-factor production in minimal media with/without serum supplementation

#### (i) MIC of NaSa

To ensure that the NaSa concentrations did not inhibit growth, the MIC of the clinical isolates, PAO1 and the QS reporter strains was determined in 50 % (v/v) serum in saline. Colonies from overnight cultures on blood agar plates were diluted to OD_546_=0.1 and further diluted 1 : 1000 in 50 % (w/v) serum to approximately 10^5^ c.f.u ml^−1^. In a flat-bottomed 96-well plate (Nunc, Denmark), 150 µl inoculum was mixed 1 : 1 with different concentrations of NaSa in 50 % (w/v) serum in saline. Final concentrations of NaSa ranged between 2 and 250 mM using two-fold dilution steps. The plate was incubated for 24 h at 35 °C under static conditions. Next, the OD_600_ was measured, and concentrations of NaSa resulting in OD_600_<0.01 were used to define the MIC. At least two of the three trials needed to match for the results to be considered valid.

#### (ii) Effect of NaSa on QS activation in minimal media

To evaluate the effect of NaSa on QS activation, reporter strains were used as described previously [41] with modifications. In brief, overnight cultures of the reporter strains *lasB::gfp*(ASV), *rhlA::gfp*(ASV) and *pqsA::gfp*(ASV) grown in TSB supplemented with 60 µg ml^−1^ gentamicin were washed twice in AB medium by 5 min centrifugation at 4000 ***g***, diluted to OD_546_=0.1 and further diluted 1 : 10 in AB medium. NaSa was diluted to 10 mM in the reporter-strain-containing AB medium and further two-fold diluted in same medium to 20 µM. A volume of 50 µl was added to the wells of a black 384-well plate with a clear bottom. Absorbance at 450 nm and bottom-read FI at an excitation of 530/20 nm and an emission of 485/30 nm were recorded every 15 min for 20 h at 35 °C in a plate reader. For each experimental group, the average AUC from triplicate wells was calculated from the FI curves (baseline- and blank-adjusted) and normalized by the OD. Average data from three independent experiments are presented.

#### (iii) Effect of NaSa on QS inhibition and virulence factor production in serum-supplemented minimal media

A checkerboard approach was used to investigate the effect of NaSa and serum on QS gene expression and virulence-factor production. In a 96-well plate, serum was added to the first column of five rows and serially diluted column-wise (1 : 2, four times) in AB medium; the fifth column contained only AB medium. Similarly, 20 mM NaSa in AB medium was added to the first row of five columns and serially diluted row-wise (1 : 10, four times); the fifth row contained only AB medium. Next, the two dilution series were mixed 1 : 1 with each other, creating a pattern with 25 unique serum-NaSa combinations. A volume of 45 µl of each combination was added to black 384-well plates with a clear bottom. Overnight cultures in 3 ml TSB of PAO1 *lasB::gfp*(ASV), PAO1 *rhlA::gfp*(ASV) or PAO1 *pqsA::gfp*(ASV) were washed twice in saline by centrifugation at 4 000 ***g*** for 5 min and resuspended in AB medium to an OD_546_ of 0.1. In total, 5 µl of the bacterial suspensions were added to the 45 µl checkerboard wells. Absorbance at 450 nm and bottom-read FI at an excitation of 530/20 nm and emission of 485/30 nm were recorded every 15 min during a 20 h incubation at 35 °C in a plate reader. All medium used contained 60 µg ml^−1^ gentamicin. The average AUC of baseline-adjusted and OD-normalized FI curves from three independent experiments was calculated. Growth curves of the reporter strains were obtained to ensure that the chosen serum and NaSa levels did not significantly inhibit bacterial growth (Fig. S1, available in the online version of this article).

The same checkerboard approach was used with *P. aeruginosa* PAO1, using the same medium but without antibiotics. After 72 h of incubation at 35 °C in a humidified incubator, cultures were centrifuged at 14 000 ***g*** for 10 min, and 50 µl supernatants were transferred to a clear 384-well plate (Nunc, Denmark). The OD was measured at 405 and 690 nm for indirect quantification of siderophores and pyocyanin production, respectively [93, 94]. For samples resulting in OD readings above the limit of detection of the plate reader, the well volumes were reduced and subsequent OD readings were pathway-corrected. Average data from three independent experiments are presented.

### Effect of NaSa on clinical *P. aeruginosa* strains from chronic wounds

Overnight cultures of strains 1, 4, 12 and PAO1 on blood agar plates were diluted to OD_546_=0.1 in LB supplemented with 50 % (w/v) serum and NaSa (0, 2 or 10 mM final concentrations) and cultured in a 24-well microtiter plate (Nunc, Denmark) at 35 °C and 100 r.p.m. for 24 h and 48 h. Cell-free LB with 50 % (w/v) serum served as a blank sample. After 24 h and 48 h, 500 µl of bacterial suspension from each well was transferred to an Eppendorf tube and centrifuged at 14 000 ***g*** for 10 min. Next, the production of the following virulence factors was evaluated.

#### (i) Pyocyanin and siderophore production

A volume of 300 µl of the supernatant was transferred to a 96-well microtitre plate (Nunc, Denmark). The presence of pyocyanin and siderophores was evaluated after 48 h of growth using a plate reader by determining their blank-corrected ODs at 405 and 690 nm, respectively. Average data from three independent experiments were calculated.

#### (ii) Alkaline protease activity

Alkaline protease activity was evaluated in an azocasein-based assay [95] after 24 h of growth. Altogether, 100 µl of supernatant was added to 400 µl 1 % (w/v) azocasein solution in 100 mM TRIS (pH 10) and incubated at 35 °C and 100 r.p.m. for 30 min before 500 µl 10 % (w/v) trichloroacetic acid was added to stop the degradation of azocasein. The tubes were centrifuged at 14 000 ***g*** for 10 min before the supernatant was mixed with 1 M NaOH in a 1 : 1 ratio. Of the mixture, 100 µl was further diluted 1 : 1 in 1 M NaOH and transferred to a 384-well microtitre plate, and OD was recorded at 420 nm using a plate reader. Data were blank-subtracted against cell-free LB supplemented with 50 % (v/v) serum. Average data from three independent experiments were calculated.

#### (iii) Elastase protease activity

Elastase activity was measured after 24 h of growth. 5-FAM fluorophore-labelled elastin (Anaspec, USA) was diluted to 1 mg ml^−1^ in dH_2_O and further diluted 1 : 30 in PBS. Then, 25 µl elastin solution was added to 25 µl supernatant diluted 1 : 10, 1 : 100 and 1 : 1000 in a black 384-well plate. Porcine pancreas elastase was diluted two-fold in PBS from 50 U ml^−1^ to 0.7 U ml^−1^ to serve as the standard curve and added to the plate in triplicate. Pure PBS served as the blank. FI was recorded every 90 s in a plate reader at 35 °C using an excitation of 530/20 nm and emission of 485/30 nm. The average blank-corrected increase in relative FI per min (r.f.u. min^−1^) from three independent experiments was calculated. Samples were chosen from dilutions resulting in data within the linear part of the standard curve, and the results were corrected for any dilutions.

#### (iv) Stapholytic activity

*Staphylococcus aureus* ATCC 25923 was cultured, harvested and boiled as described above, but without the addition of TSA. The boiled cells were diluted in 10 mM Na_2_PO_4_ (dH_2_O) until OD_521_=0.1. Overall, 180 µl of *S. aureus* solution was added to a 96-well plate (Nunc, Denmark) prior to the transfer of 20 µl supernatant from 24 h old cultures to the wells. The OD at 600 nm was recorded every 30 s for 30 min in a plate reader at 35 °C. The average decrease in the blank-corrected OD per min during the first 10 min was calculated from three independent experiments.

#### (v) Biofilm formation

Biofilm formation was evaluated as described previously using strains 1, 4, 12 and PAO1 cultured in saline supplemented with 50 % (v/v) serum and 0, 1 or 10 mM NaSa. The data are presented as blank-corrected average OD values from three independent experiments, each with three technical replicates.

### Effect of serum on selected virulence factors in clinical *P. aeruginosa* strains from chronic wounds

The effect of serum on the production of siderophores and pyocyanin was investigated in LB with and without the addition of 50 % (v/v) serum after 24 and 48 h cultures, respectively. Overnight cultures of strain 1–14 and PAO1 in TSB were washed twice by centrifugation at 4000 ***g*** for 10 min and resuspended in saline to OD_546_=0.1. In total, 5 µl of these bacterial suspensions were added to 195 µl LB or LB supplemented with 50 % (v/v) serum in 96-well microtitre plates. After incubation at 35 °C in a humidified incubator, 50 µl of bacterial supernatants of each sample were collected by centrifugation at 4000 ***g*** for 10 min and transferred to a 384-well plate (Nunc Denmark). Siderophore and pyocyanin production was evaluated by absorbance measurements at 405 nm and 690 nm, respectively. The experiment was repeated three times. Biofilm formation was studied as described before, but using LB with or without 50 % (v/v) serum as media; *N*=6.

### Statistics

One-way ANOVA followed by two-sided Dunnett’s post hoc tests was performed to evaluate significant differences between the control (no treatment) and each of the different concentrations of NaSa and between the control (no serum) and each of the different concentrations of serum in medium with regard to the expression of *lasB::gfp*, *rhlA::gfp* and *pqsA::gfp* genes, production of siderophores, pyocyanin, elastase, alkaline protease, stapholytic protease and biofilm. Two-sided Student’s *t*-tests were performed to calculate differences in pyocyanin, siderophores and biofilm production between the control (no serum) and the serum-containing medium as well as for the production of rhamnolipids between the strains and the blank. Statistical analyses were performed using SPSS Statistics 21 (IBM Corporation, USA) with a significance level set at *P*<0.05.

## Supplementary Data

Supplementary material 1
