# Supplementary material for: Sodium salicylate interferes with quorum-sensing-regulated virulence in chronic wound isolates of Pseudomonas aeruginosa in simulated wound fluid
Source: J Med Microbiol. 2020 Apr 22;69(5):767–80. doi: 10.1099/jmm.0.001188 (PMC7451038; doi:10.1099/jmm.0.001188)
Supplement: Supplementary material 1 [file jmm-69-767-s001.pdf]

Supplementary Material

Sodium salicylate interferes with quorum sensing-regulated virulence in chronic wound isolates of *Pseudomonas aeruginosa* in simulated wound fluid

Erik Gerner[1,2,3,#], Sofia Almqvist[2], Maria Werthén[1,3,4], and Margarita Trobos[1,3,#]

[1]Department of Biomaterials, The Sahlgrenska Academy at University of Gothenburg, Gothenburg, Sweden

[2]Mölnlycke Health Care AB, Gothenburg, Sweden

[3]Center for Antibiotic Resistance Research (CARe) at University of Gothenburg, Gothenburg, Sweden

[4]Department of Health Sciences, University West, Trollhättan, Sweden

[#]Corresponding authors:

Erik Gerner

and

Margarita Trobos (ORCiD: 0000-0002-9253-6771)
margarita.trobos@biomaterials.gu.se

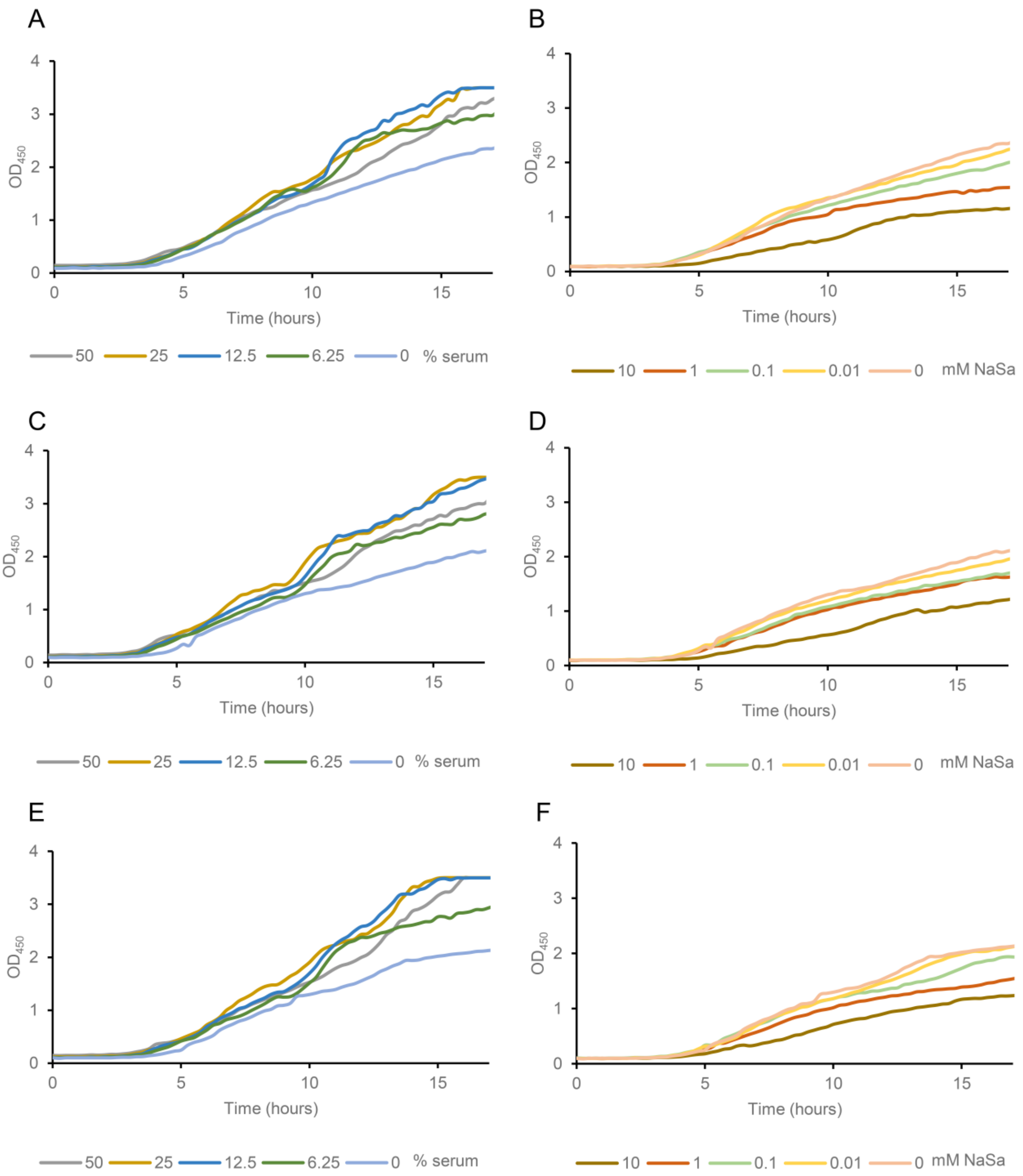

Supplementary Figure S1 Growth curves of PAO1 reporter strains in AB medium supplemented with serum (0-50%) and treated with NaSa (0-10 mM): (A-B) lasB::gfp, (C-D) rhlA::gfp and (E-F) pqsA::gfp in medium with serum (A, C and E) or NaSa (B, D and F). Each curve represents average data from three independent experiments.

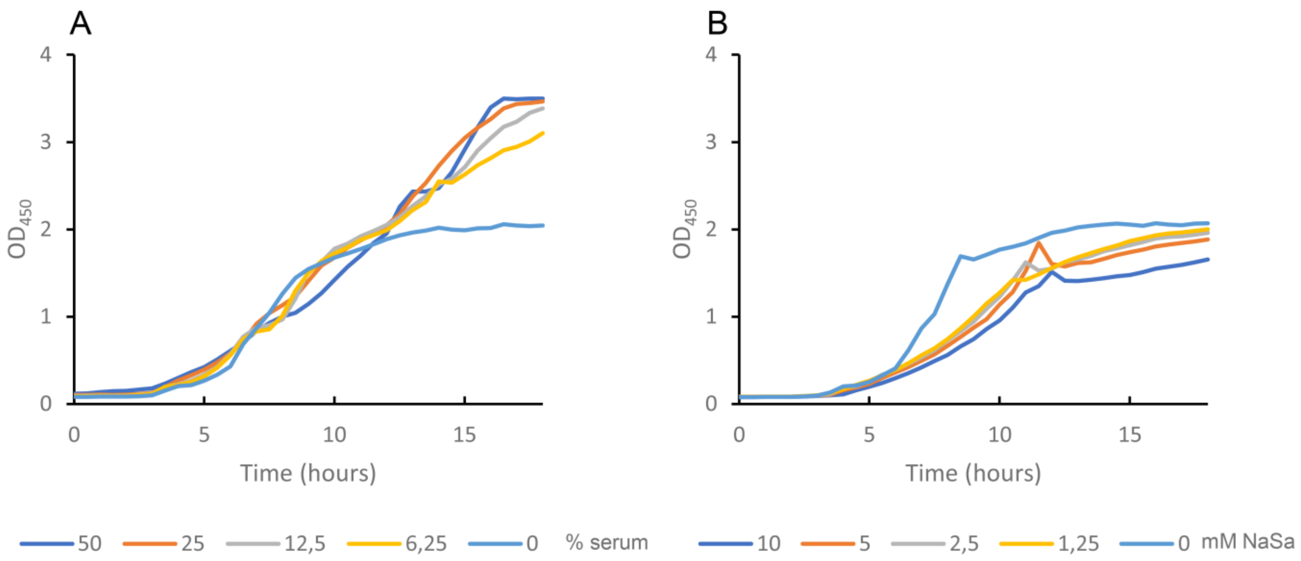

Supplementary Figure S2 Growth curves of PAO1 in AB medium supplemented with serum or treated with NaSa. (A) 0-50% serum (B) 0-10 mM NaSa. Each curve represents average data from three independent experiments.

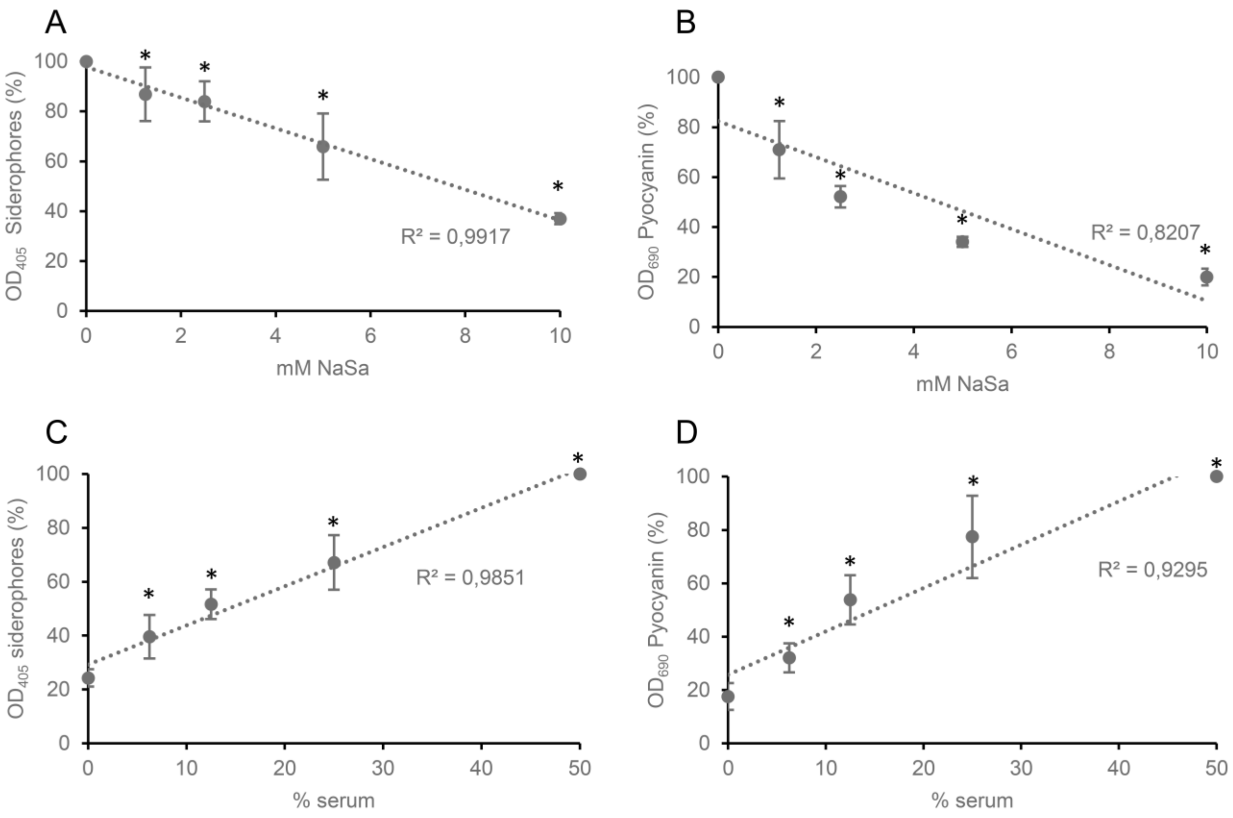

Supplementary Figure S3 Effect of NaSa and serum on PAO1 pyocyanin and siderophore production after 72 h of growth. (A) and B) Effect of NaSa on siderophore and pyocyanin production in 50% serum. (C) and (D) Effect of serum on siderophore and pyocyanin production in 0 mM Nasa, respectively. One hundred percent corresponds to OD 6.85 (A and C) and 1.91 (B and D) for siderophores and pyocyanin, respectively. Each data point represents the average data from three independent experiments. Error bars represent ± SD. * Indicates a statistically significant difference compared to 0 mM NaSa (A-B) and to 0% serum (C-D) with a p-value <0.05 and N=3 based on one-way ANOVA Dunnett´s post hoc test.

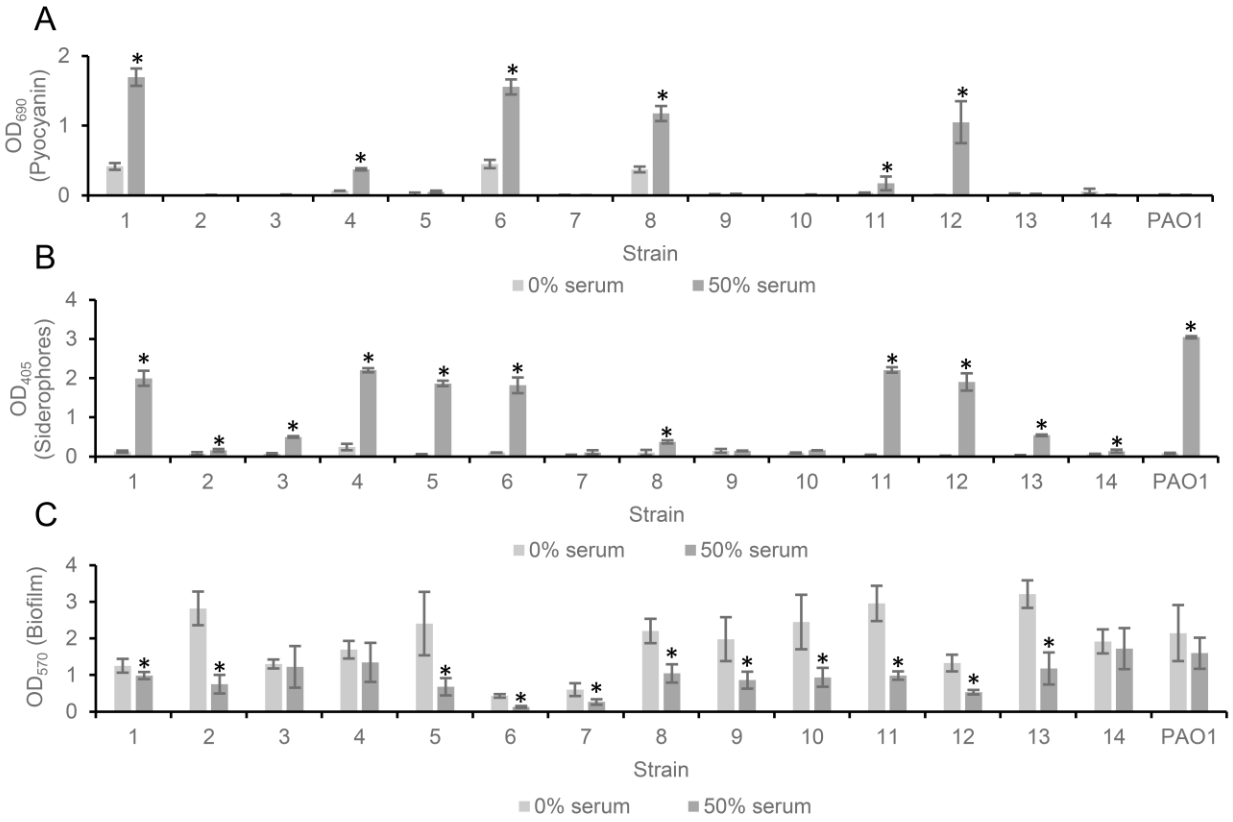

Supplementary Figure S4 Effect of serum on selected virulence factors in clinical wound strains of *Pseudomonas aeruginosa*. (A) Pyocyanin and (B) siderophore production in LB ± 50% serum after 48 and 24 h of growth, respectively. (C) Biofilm biomass produced by the strains on polystyrene pegs after 48 h of growth under dynamic conditions in TSB ± 50% serum as measured by crystal violet OD. Data are based on three (A and B) or six (C) independent experiments. * Indicates a statistically significant difference between serum and control for each strain virulence factor with a p-value < 0.05 using Student's two-tailed t-test.

Table S1. Minimum inhibitory concentration of sodium salicylate for *Pseudomonas aeruginosa* strains cultured in 50% serum in saline.

**MIC = 62.5 mM**	Strain 7, 8, 9
**MIC = 125 mM**	Strain 1-6, 10-14, PAO1, PAO1 P*lasB*::*gfp(ASV)*, PAO1 *rhlA*::*gfp(ASV)*, PAO1 *pqsA*::*gfp*
